# Antibacterial FANA oligonucleotides as a novel approach for managing the Huanglongbing pathosystem

**DOI:** 10.1038/s41598-021-82425-8

**Published:** 2021-02-02

**Authors:** Andrés F. Sandoval-Mojica, Wayne B. Hunter, Veenu Aishwarya, Sylvia Bonilla, Kirsten S. Pelz-Stelinski

**Affiliations:** 1 Department of Entomology and Nematology, University of Florida, Citrus Research and Education Center, IFAS, Lake Alfred, FL 33850-2299 USA; 2 Agricultural Research Service, U.S. Department of Agriculture, Fort Pierce, FL 34945 USA; 3 AUM LifeTech, Inc., Philadelphia, PA 19104 USA

**Keywords:** Antimicrobials, Pathogens, Entomology

## Abstract

*Candidatus* Liberibacter asiaticus (*C*Las), a bacterium transmitted by the Asian citrus psyllid, *Diaphorina citri*, is the causal agent of citrus greening disease, or Huanglongbng (HLB). Currently, vector population suppression with insecticides and tree removal are the most effective strategies for managing the HLB pathosystem. In this study, we assessed the bactericidal capabilities of 2′-deoxy-2′-fluoro-d-arabinonucleic acid antisense oligonucleotides (FANA ASO) both in vitro and in vivo by (1) confirming their capacity to penetrate insect cells, (2) silencing bacterial essential genes, and (3) quantifying reductions in bacterial titer and *D. citri* transmission. We confirmed that FANA ASO are able to penetrate insect cells without the use of a delivery agent. Expression of an essential gene in the *D. citri* endosymbiont, *Wolbachia* (wDi), significantly decreased by 30% following incubation with a wDi-specific FANA ASO. Viability of isolated wDi cells also decreased in response to the FANA ASO treatment. Delivery of a *C*Las-specific FANA ASO to infected adult *D. citri* in feeding assays resulted in significant silencing of a *C*Las essential gene. *C*Las relative density and transmission were significantly lower among *D. citri* fed FANA ASO in diet compared to untreated insects. Root infusions of a *C*Las-specific FANA ASO into infected *Citrus* trees significantly reduced *C*Las titer during a 30-day trial. Our results suggest that FANA ASO targeting insect-transmitted plant bacteria or insect endosymbionts may be useful tool for integrated management of agricultural pathogens.

## Introduction

Citrus greening disease, or Huanglongbing (HLB), is a devastating disease affecting citrus groves worldwide^1^. HLB is characterized by symptoms such as leaf chlorosis, undeveloped fruit, premature fruit drop, and twig die-back, culminating in tree death^1, 2^. The HLB causal agent in Asia, North America and Brazil is “*Candidatus* Liberibacter asiaticus” (*C*Las), a phloem-limited Alphaproteobacterium^3^ transmitted by the Asian citrus psyllid, *Diaphorina citri* Kuwayama (Hemiptera: Liviidae)^4^.

*C*Las is acquired by the psyllid while feeding on the phloem of infected plants. Once inside the digestive tract of the insect, the bacterium can colonize and propagate. Nevertheless, *C*Las must pass through the gut wall into the hemolymph and reach the salivary glands before it can be successfully transmitted^4^. Besides *C*Las, *D. citri* harbors three known bacterial endosymbionts: “*Candidatus* Carsonella ruddii”, a Gammaproteobacterium which may provide nutritional benefits^5, 6^; “*Candidatus* Profftella armatura”, a Betaproteobacterium with a putative defensive role^7^; a strain of the Alphaproteobacteria *Wolbachia* (wDi), which is widely distributed amongst insect species^8, 9^. *D. citri* endosymbionts represent a potentially important target for bactericidal control strategies developed for *C*Las management due to their essential contributions to host fitness.

Current management of HLB focuses on controlling *D. citri* populations by spraying insecticides such as pyrethroids, organophosphates, and neonicotinoids, on a calendar basis^10–12^. However, the prevalent use of insecticides has led to the development of resistance among *D. citri* populations^13–15^. Development of alternative tools for *D. citri* management is therefore necessary to reduce the impact of HLB. Alternative control includes the application of antimicrobial compounds in combination with traditional insecticidal programs to disrupt pathogen acquisition by the vector and population numbers, respectively. Antimicrobial treatments for *C*Las may indirectly effect *D. citri* by altering pathogen transmission and endosymbiont titers. Given their reliance on endosymbionts for nutrition, reduction of symbionts following antibiotic exposure may negatively affect *D. citri* fitness^16, 17^. Problematically, antibiotics are non-specific and must be ingested in sufficient quantities to reduce or eliminate *C*Las in infected *Citrus* plants and psyllid vectors. Targeted approaches, such as double stranded RNA (dsRNA)-mediated gene silencing (RNAi), show great promise for *D. citri* management^18–20^; however, targeting of *C*Las and *D. citri* endosymbionts with dsRNA is complicated by the absence of RNAi in bacteria.

The recent development of new antisense technologies to obtain silencing of essential bacterial genes offers a possible opportunity for HLB management.

FANA antisense oligonucleotides (2′-deoxy-2′-fluoro-d-arabinonucleic acid, herein referred to as FANA ASO) are synthetic single-stranded nucleic acid analogs that can modulate gene expression by enzymatic degradation of a target RNA^21–23^. FANA ASOs recognize and bind to specific RNA forms, including mRNAs, miRNAs, and long noncoding RNAs, through complementary base pairing. Contrary to the RNAi pathway that requires the interaction of several enzymes^24, 25^, FANA ASOs utilize RNase H-mediated RNA cleavage^26^. FANA ASO first binds an RNA target by Watson–Crick base paring^27^. Ribonuclease H (RNase H), an endogenous enzyme present in both prokaryotes and eukaryotes, recognizes the FANA/mRNA duplex and cleaves the RNA within the hybrid^28, 29^. Following cleavage, the fragmented mRNA is further degraded by nucleases and the FANA ASO are recycled within the cell; the degradation of multiple mRNA copies by a single FANA ASO increases the silencing efficiency and lowers the dose required^22, 30^. FANA ASOs are characterized by a phosphorothioate backbone and modified flanking nucleotides, in which the 2′-OH group of the ribose sugar was substituted by a fluorine atom. These chemical modifications increase the resistance of the FANA ASO to degradation and enhance binding to targeted mRNA^30, 31^.

In this study, we designed and characterized FANA ASOs the targeting bacteria associated *D. citri*: the citrus pathogen, *Ca*. Liberibacter asiaticus, and *Wolbachia*. The FANA ASO were evaluated in vitro and in vivo in order to confirm penetration of insect host cells, silence bacterial essential genes, and assess bactericidal activity. Together, our results indicate the potential for FANA ASO use as part of current HLB management strategies.

## Results

### FANA ASO penetration of insect cells

**Figure 1:**
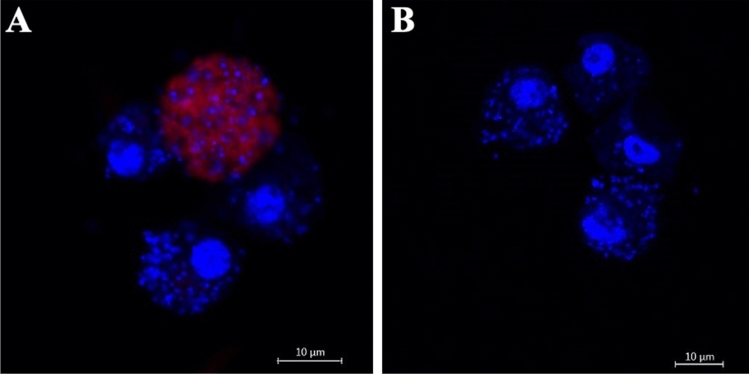
Localization of FANA oligo in S2-wDi cells. Images were acquired using DAPI (blue) and Alexa Fluor 647 (red) filters. (**A**) Cells incubated with 10 µM of a FANA ASO tagged with Alexa Fluor 647. (**B**) Untreated cells. DAPI stained the S2 cells nuclei as well as wDi chromosomes (small blue dots).

Penetration of insect cells by FANA ASO was evaluated by incubating a suspension of S2-wDi cells with a fluorescently labeled oligonucleotide for 24 h. Labeled FANA ASO was detected inside S2-wDi cells (Fig. 1A) within one day post-treatment without the use of a transfection agent. No signal was detected in untreated S2-wDi cells (Fig. 1B).

### gyrA-FANA effect on Wolbachia cell culture

**Figure 2:**
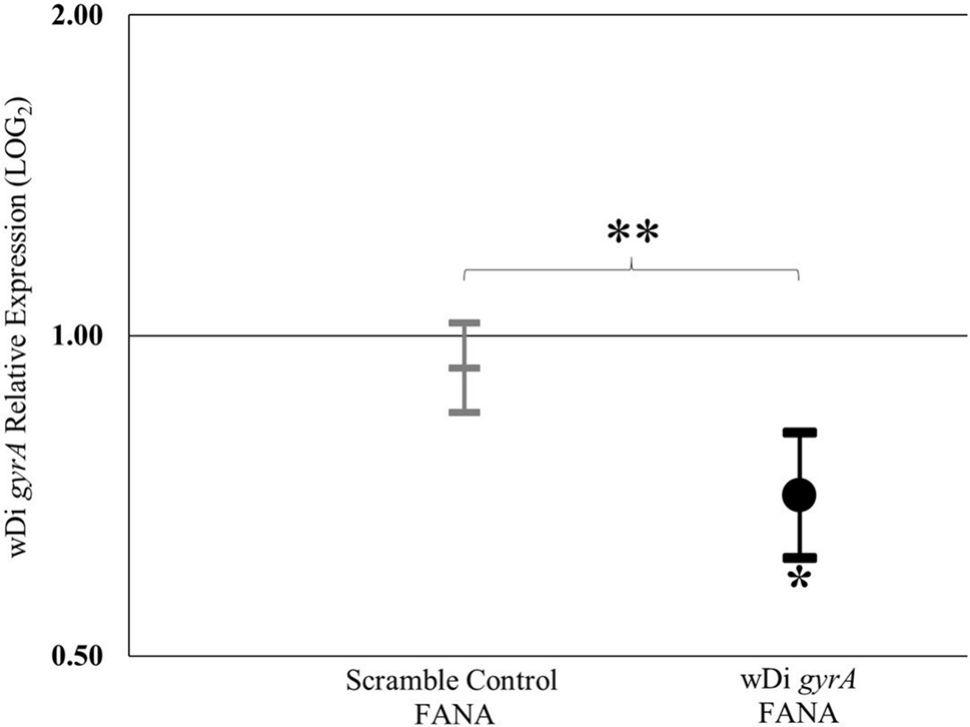
Effect of FANA ASO on mRNA levels of wDi *gyrA* in cell culture. The markers represent the expression of the target gene in the experimental condition compared to its expression in the untreated condition. Values lower than 1 indicates gene suppression. Results are means of triplicate experiments ± SE. *P ≤ 0.05; **P ≤ 0.001 (TIBCO Statistica, v. 13.3.0: https://www.tibco.com/products/data-science).

To examine the competence of FANA ASO to mediate the degradation of specific bacterial genes in cultured insect cells, S2-wDi cells were incubated with 5 µM of a FANA oligo complementary to the mRNA of wDi *gyrA* gene (*gyrA*-FANA). As a negative control, a group of cells were exposed to a FANA oligo designed with a scrambled sequence not targeting any wDi gene (scramble control FANA; SC-FANA). Compared to untreated cells, the *gyrA*-FANA treatment significantly reduced the amount of the target mRNA by 30% [*t* (16) = − 2.60, *p* = 0.019] (Fig. 2). The wDi *gyrA* transcript level was significantly lower in the cells exposed to *gyrA-*FANA, compared to cells treated with the scramble control [*t* (16) = 2.98, *p* = 0.009]. No significant difference in the expression of the wDi *gyrA* gene was observed between cells incubated with 5 µM of the scramble control FANA and untreated cells [*t* (16) = − 0.65, *p* = 0.52].

**Figure 3:**
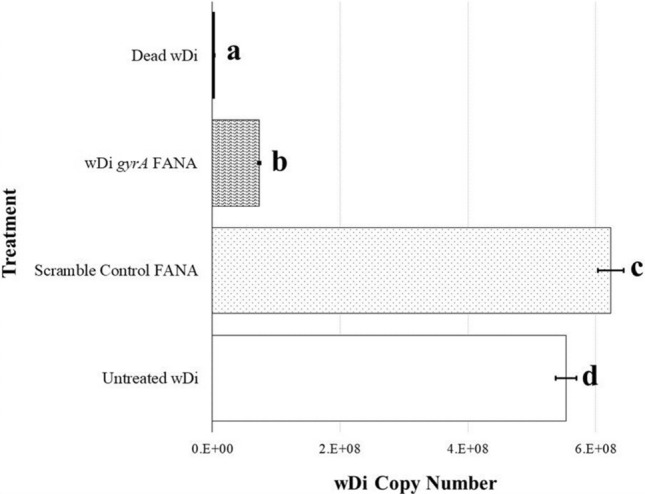
Density of viable wDi cells after incubation with 5 µM FANA solutions. Dead wDi refers to bacterial cells that were incubated at 95 °C for 10 min. Statistical differences between the treatments (*P* ≤ 0.05) are designated by different letters (ANOVA: *F* (3, 8) = 614.35; *P* ≤ 0.0001). Results are means of triplicate experiments ± SE (TIBCO Statistica, v. 13.3.0: https://www.tibco.com/products/data-science).

In a second experiment, wDi endosymbionts in *Drosophila* S2 cells were treated with *gyrA*-FANA in a bacterial viability assay to evaluate the ability of ASOs to inhibit *Wolbachia* replication. We observed a 88% and 87% reduction in viable wDi in cells treated with *gyrA*-FANA ASO when compared with SC-FANA or non-treated cells, respectively (Fig. 3). This suggests that inhibition of DNA gyrase subunit A gene expression is lethal for the *Wolbachia* cells. The high wDi copy number in cells treated with the SC-FANA indicates that the FANA ASO alone did not have non specific effects on the bacterial cells.

### CLas LigA silencing in *D. citri*

**Figure 4:**
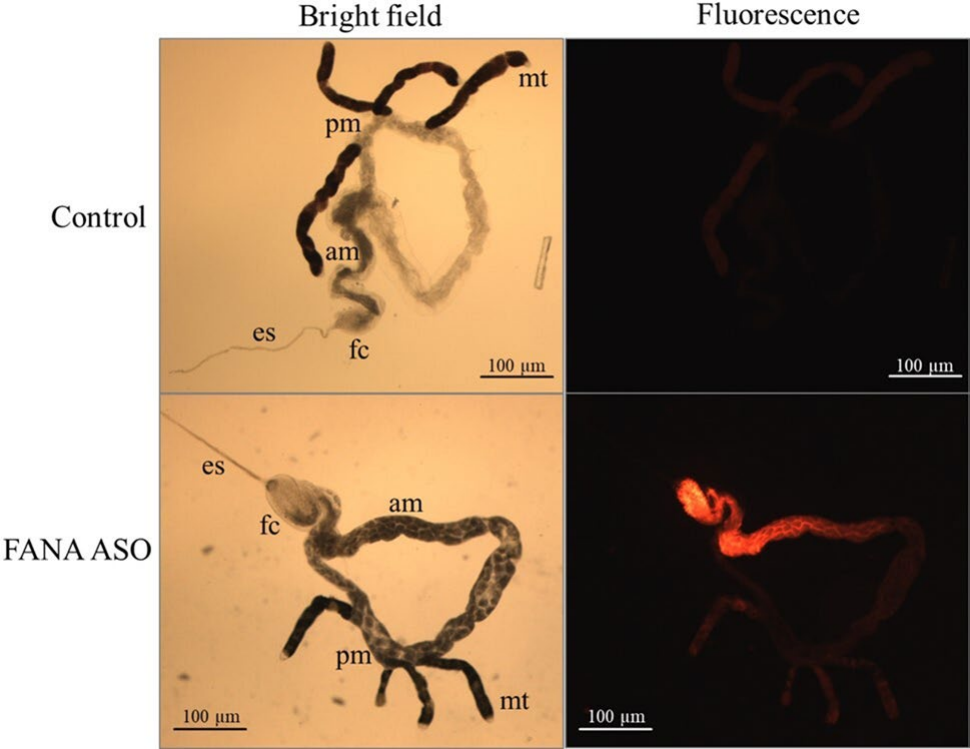
Localization of FANA oligo in the alimentary canal of *D. citri* adults. Psyllid guts were dissected and imaged after feeding on *Citrus* leaves previously treated with a 5 µM solution of an Alexa Fluor 594 labeled FANA ASO. *es* esophagus, *fc* filter chamber, *am* anterior midgut, *pm* posterior midgut, *mt* Malpighian tubule.

To evaluate ingestion of FANA ASOs from the vascular system of *Citrus* plants, adult *D. citri* were enclosed on ‘Pineapple’ sweet orange leaves which had previously absorbed a solution containing a fluorophore labelled FANA ASO. FANA ASO was detected by fluorescent microscopy in the alimentary canals of *D. citri* after 48 h of feeding. Oligonucleotides were detected mainly in the psyllid esophagus, filter chamber, and anterior midgut (Fig. 4).

**Figure 5:**
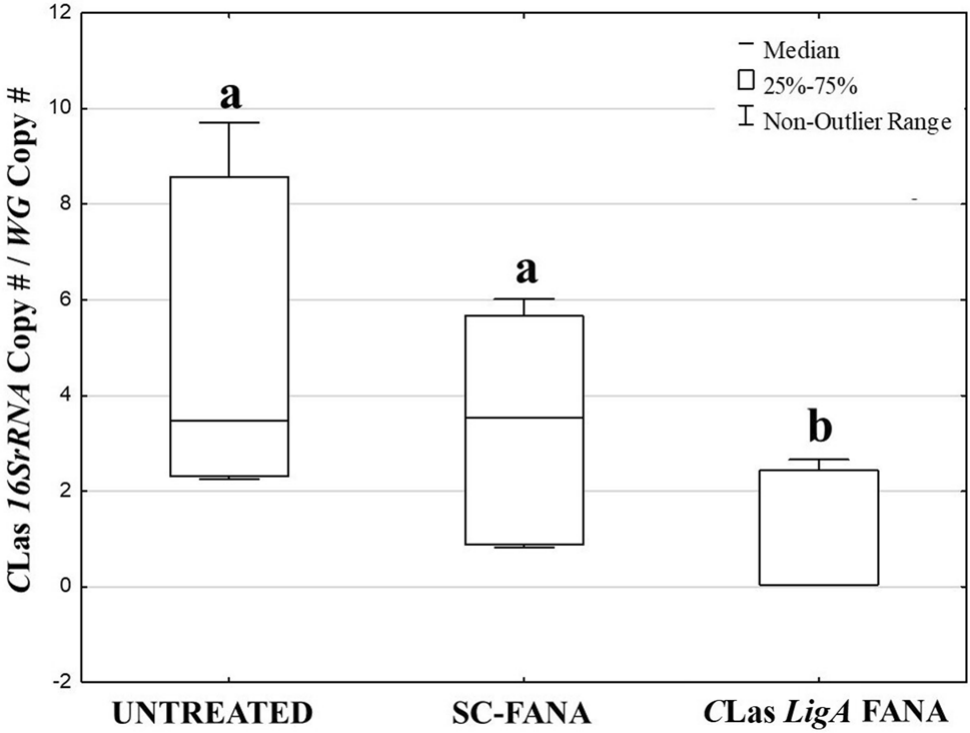
Effect of FANA ASO on mRNA levels of *C*Las *LygA* in *D. citri* adults. The markers represent the expression of the target gene in the experimental condition compared to its expression in the untreated condition. Values lower than 1 indicates gene suppression. Results are means of triplicate experiments ± SE. **P ≤ 0.001 (TIBCO Statistica, v. 13.3.0: https://www.tibco.com/products/data-science).

The efficacy of FANA ASOs in silencing the expression of *C*Las genes was tested by delivering oligonucleotides to *D. citri* adults through the vascular system of excised *Citrus* leaves. The leaves were placed in a 5 µM FANA solution of *LigA*-FANA, which targets the *C*Las *LigA* transcript, SC-FANA, or water. After seven days of continuous feeding, we observed a 75% decrease in the expression of *C*Las *LigA* gene in *D. citri* following *LigA-*FANA treatment as compared with untreated psyllids [*t* (16) = − 3.18, *p* = 0.006] (Fig. 5). Expression of *C*Las *LigA* was also significantly lower in psyllids exposed to *LigA-*FANA compared to insects treated with the SC-FANA [*t* (13) = 3.08, *p* = 0.009]. There was no significant difference in *C*Las *LigA* mRNA expression between psyllids fed SC-FANA and untreated psyllids [*t* (13) = − 0.007, *p* = 0.99].

### *LigA-*FANA effect on *C*Las titer in *D. citri* and *Citrus*

**Figure 6:**
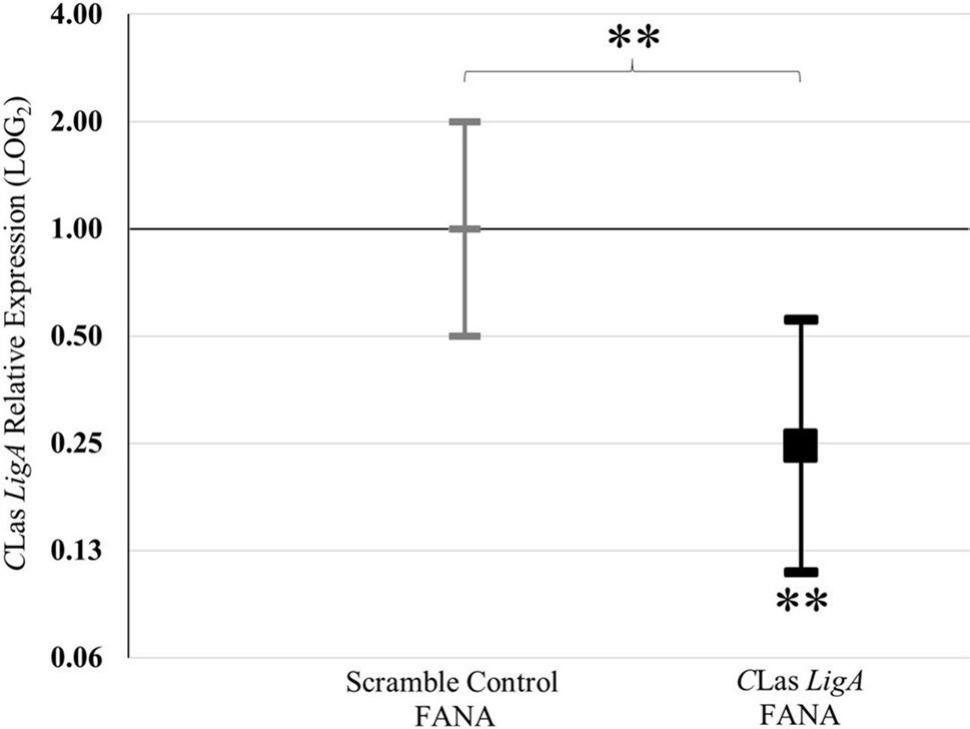
Distribution of *C*Las titer in *D. citri* adults after feeding 5 µM FANA solutions for seven days. Statistical differences between the treatments (*P* ≤ 0.05) are designated by different letters (KW-H [2, 27] = 10.6984, *p* = 0.0048). Results are medians of triplicate experiments.

*D. citri* adults were fed an artificial diet containing *LigA*-FANA at a concentration that significantly reduced the expression of the *C*Las *LigA* gene. Seven days post-treatment, *C*Las density was significantly lower among *D. citri* that ingested *LigA-*FANA, as compared with psyllids that were untreated or fed SC-FANA (Fig. 6). No difference in *C*Las copy number was observed between *D. citri* adults exposed to the SC-FANA-treated and untreated psyllids. The results demonstrated that degradation of *C*Las *LigA* mRNA significantly reduced bacerial density.

**Figure 7:**
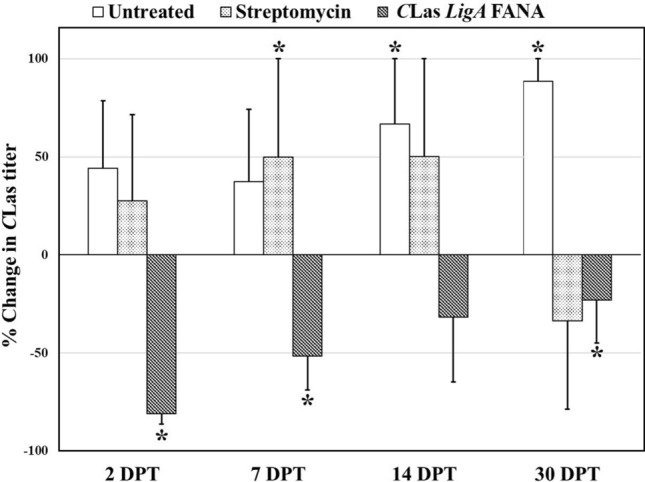
Effect of FANA ASO on *C*Las titer in *Citrus* plants. Treatment effect was expressed as percentage change in *C*Las titer [(mean titer after treatment − mean titer prior to treatment)/mean titer prior to treatment] × 100. Means and standard errors are represented by bars and whiskers, respectively. Bars followed by an asterisk indicates significant change (increase or decrease) in *C*Las copy number as compared to that before treatment (DPT: days post treatment) (TIBCO Statistica, v. 13.3.0: https://www.tibco.com/products/data-science).

Root-applied *LigA* FANA significantly reduced the quantity of the *C*Las in infected *Citrus* trees (Fig. 7). The suppression effect was persistent during the 30 days sampling period and the highest Liberibacter repression was observed two and seven days post treatment (81% and 51.6%, respectively). Root infusion application of streptomycin sulfate into infected *Citrus* plants did not significantly reduce the *C*Las titer. *C*Las titers significantly increased by 67 and 89% in *Citrus* plants infused with water after 14 and 30 days, respectively (Fig. 7).

### Effect of *LigA-*FANA on CLas transmission

**Figure 8:**
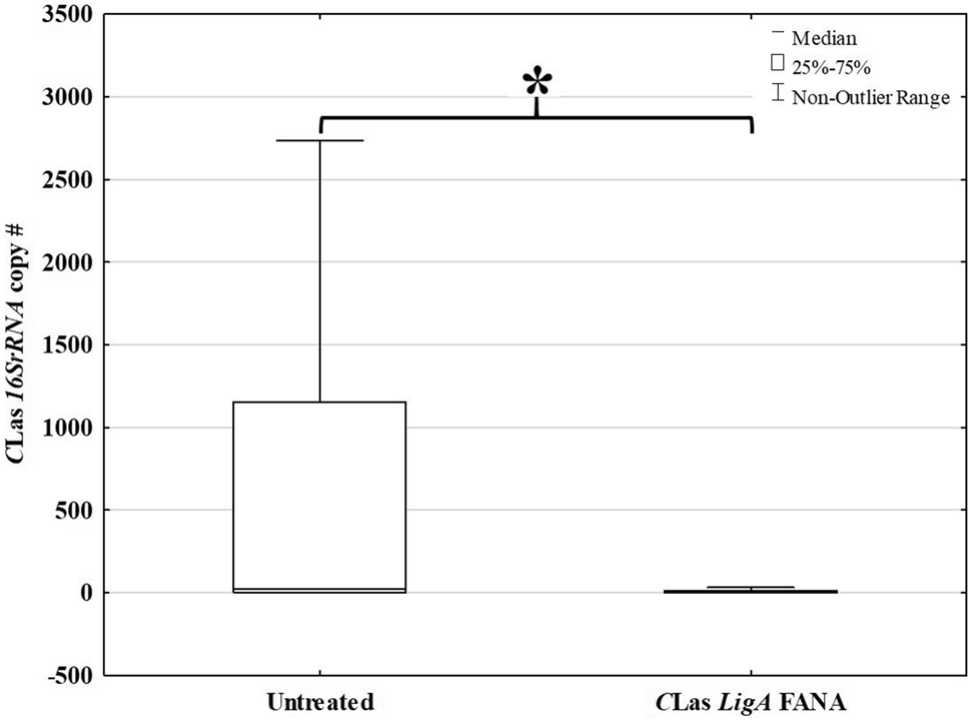
*C*Las titer detected in *Citrus macrophylla* seedlings following exposure to untreated or FANA-treated infected adult *D. citri*. Statistical differences between the treatments are designated by *(*P* ≤ 0.05) (TIBCO Statistica, v. 13.3.0: https://www.tibco.com/products/data-science).

Fewer *C*Las infections occurred in *Citrus* following exposure to *D. citri* treated with FANA ASO prior to transmission feeding. Plants exposed to *D. citri* treated with *LigA*-FANA had significantly fewer *C*Las gene copies as compared to citrus plants that were exposed to the untreated psyllids (*U* = 720, n1 = n2 = 45, *p* = 0.018) (Fig. 8).

## Discussion

This study demonstrated the efficacy of FANA ASO in reducing the amount of bacterial mRNA, in both in vitro and in vivo experiments, causing a reduction in bacterial titer. Specifically, we determined that the copy number of “*Candidatus* Liberibacter asiaticus” declined in *D. citri* adults following oral ingestion of FANA ASO. We targeted a specific region of the *C*Las *LigA* gene and confirmed gene silencing. These results support the potential of FANA ASOs as an environmentally friendly alternative for the HLB management, similar to RNAi. Both FANA ASOs and RNAi are post-transcriptional gene silencing technologies that involve the binding of complementary oligonucleotides to target RNA through base paring^21^. However, RNAi can only be used to target eukaryotes and some fungi, due to the pathway of genes present for insect fitness or survival^18–20^ but cannot target *C*Las directly because bacteria lack a homologous RNAi pathway. In addition, intrinsic characteristics of FANA ASO may position them as a better choice than RNAi for controlling HLB. Double stranded RNA (dsRNA) persistence has been a major challenge in RNAi-based pest control^32^. For example, degradation of dsRNA has been reported in hemipteran species such as *Lygus lineolaris* (Palisot de Beauvois) (Hemiptera: Miridae) and *Acyrthosiphon pisum* (Harris) (Hemiptera: Aphididae), where the gut pH conditions and possible nuclease enzymes in the saliva and hemolymph digested the exogenous ribonucleic acid^33, 34^. The stability of FANA ASOs to hydrolysis under acidic and basic conditions is comparatively greater than that of DNA or RNA^30^. In addition, FANA ASO display resistance to the action of both endo and exonucleases^30, 35, 36^ due to the phosphorothioate backbone and a 2′-deoxy-2′-fluoro-beta-d-arabinose furanose modification. FANA ASO resistance to degradation is expected to produce more effective silencing of insect genes than that achieved by dsRNA because the former will persist in host cells.

In this study, we demonstrated that FANA oligonucleotides are capable of penetrating *Drosophila* S2 cells infected with wDi without a transfection agent or molecular conjugation. In a subsequent in vitro experiment, downregulation of the wDi *gyrA* gene by a complementary FANA ASO suggests that the oligonucleotide also penetrated the bacterial cells. Self-delivery of FANA ASOs was previously reported by Souleimanian and colleagues^37^ using a human prostate cancer cell line, who observed that FANA ASOs effectively silenced the expression of the Bcl-2 protein in the absence of any carriers or conjugation. The self-delivery property of FANA oligos may be explained by the phosphorothioate modification in their backbone, which has a high affinity for proteins commonly occurring on the cell surface membrane, therefore promoting adsorptive endocytosis^38^.

Oral delivery of *LigA-*FANA to adult *D. citri* decreased *C*Las *LigA* expression and reduced *C*Las density in infected psyllids. *C*Las is found throughout the tissues of infected *D. citri*^39–41^; however, the relative copy number of *C*Las genome is significantly higher in the alimentary canal than in with the rest of the insect body, suggesting that *C*Las may replicate or accumulate in the digestive tract of *D. citri*^40^. Concentrated localization of *C*Las in the *D. citri* digestive tract could facilitate bacterial penetration and binding to a homologous mRNA by ingested FANA ASOs.

The recommended control strategy for HLB involves the use of insecticides to reduce *D. citri* populations, supplemented with removal of infected trees to impede bacterial acquisition by uninfected psyllids and the creation of pathogen-free nurseries^12^. Nevertheless, insecticide resistance is a major problem for this approach^42^. Although novel tactics have been successful under greenhouse and field settings, such as thermotherapy^43^, antibiotics applications^44^ or trunk injections of plant activators^45^, there is a pressing need to find better alternatives to boost the *in planta* HLB control arsenal. Delivery of *LigA* FANA to infected *Citrus* trees through root infusions caused a significant reduction of *C*Las titer that persisted for 30 days. Reductions of pathogen titer in both its host and vector and reduced *C*Las transmission demonstrates the potential of FANA ASOs as a novel tool for HLB management.

Field verification at proportionally equivalent dosages per crop area compared to those investigated here under controlled conditions should be conducted to confirm commercial viability of FANA ASOs. In this study, only one mRNA sequence was targeted; however, FANA ASOs can be multiplexed to achieve greater silencing and increase efficacy against bacterial targets. This strategy also decreases the likelihood of resistance development, particularly when combined with multiple tools as part of an integrated management plan.

## Methods

### FANA antisense oligonucleotides

FANA antisense oligonucleotides were designed and synthesized by AUM BioTech, LLC (Philadelphia, PA). For in vitro experiments, a FANA ASO was designed to be complementary to the wDi DNA gyrase subunit A gene (*gyrA*). The *C*Las NAD-dependent DNA Ligase gene (*LigA*), was selected as the target gene in the in vivo experiments. wDi was targeted in the in vitro experiments, due to *C*Las uncultivability*.* The sequences of FANA ASOs used in this study are described in Table 1.

**Table 1:** Sequences of FANA ASO used in this study.

Target species	Target gene	Name	Sequence (5′–3′)
*Wolbachia*	*gyrA*	*gyrA-*FANA	TGGTATGGCAACCAATATTCC
*Candidatus* Liberibacter asiaticus	*LigA*	*LigA-*FANA	CGGCACAGCAATTAGAACGTC
None	None	SC-FANA	ATATCCTTGTCGTATCCCAGT

### Cell culture

*Drosophila* Schneider 2 (S2) cells were cultured in Schneider’s Drosophila medium supplemented with 10% heat-inactivated fetal bovine serum (FBS) and 1% penicillin (50U/mL)/streptomycin (50 µg/mL) at 28 °C in a non-humidified incubator. The cells were subcultured to a final density of approximately 1 × 10^6^ cell/mL every three days. Infection of S2 cells with wDi (S2-wDi cells) was accomplished as described in Bonilla et al*.* (under review).

### *D. citri* cultures

*Diaphorina citri* used in this study were obtained from a culture reared at the University of Florida Citrus Research and Education Center (CREC) (Lake Alfred, FL). The culture was established in 2005 from a field population collected in Polk Co., FL (28.0′ N, 81.9′ W), before the detection of HLB in the state. Psyllids not harboring *C*Las were maintained on uninfected ‘Pineapple’ sweet orange [*Citrus sinensis* (L.) Osb. (Rutaceae)] plants in a greenhouse not exposed to insecticides. Insects infected with *C*Las were collected from a subset of the uninfected *D. citri* culture but reared instead in *C*Las-positive ‘Pineapple’ sweet orange plants at a secure quarantine facility. Psyllids from the infected colony were routinely sampled by qPCR to confirm the presence of the bacterium. Both psyllid colonies were maintained at 26 ± 1 °C, 60–80% relative humidity and a photoperiod of 16:8 (L:D) h. To confirm the absence/presence of the bacterium in the colonies, random subsamples of both plants and insects were tested monthly using a quantitative real-time polymerase chain reaction procedure, previously described^46^.

### Localization of fluorescently labeled FANA ASOs in S2-wDi cells and *D. citri* adults

S2-wDi cells were diluted from a density of 8–10 × 10^6^ cells/mL and 90% viability to a density of 2.75 × 10^5^ cells/mL. The cells were incubated with 10 µM of a FANA ASO, designed with a scrambled sequence not targeting any wDi gene (scrambled control FANA; SC-FANA) and conjugated to Alexa Fluor 647 at its 5′ end, at 28 °C. After 24 h, 15 µL of S2-wDi cell suspension was applied into a coverslip previously covered with concanavalin A and allowed to attach for one hour. The cells were fixed with 4% paraformaldehyde during 45 min, followed by three washes with PBS of five min each at room temperature. The cells were mounted in microscope slides with VECTASHIELD with DAPI (Vector Labs, Burlingame, CA) and viewed under a confocal microscope (Leica SP8 laser-scanning Confocal).

Mature leaves from ‘Pineapple’ sweet orange trees grafted on ‘Swingle’ Citrumelo rootstocks [*Citrus paradisi* MacFaden X *Poncirus trifoliata* (L.) Raf.] were excised and placed in lidless PCR tubes with 200 µL of a FANA ASO solution in water, at a 5 µM concentration, designed with a scramble sequence and tagged with Alexa Fluor 594 at its 5′ end. The leaves were left in the solution for 24 h before placing them inside a 50 mL Falcon tube with five *D. citri* adults. After 48 h, the digestive tracts of the psyllids were dissected in PBS, placed on a microscope slide and viewed under a fluorescent microscope (Olympus BX61 Epifluorescence microscope).

### Silencing of bacterial genes with FANA ASOs

#### Cell culture assays

S2 cells infected with wDi were seeded at a density of 2.75 × 10^5^ cells/mL, in 24-well culture dishes, 24 h prior to the treatment with the FANA ASO. The cells were incubated with 5 µM of either the FANA ASO designed to target the wDi *gyrA* gene or a scrambled control FANA (SC-FANA), which was used as a negative control. The insect cells were incubated with the FANA ASO for 7 days at 28 °C. Each treatment (*gyrA*-FANA, SC-FANA and untreated control) was replicated three times.

#### *D. citri* assays

Leaves (leaf blade and petiole) were collected from uninfected Citrumelo (*C. paradisi* X *P. trifoliata*) trees, washed in 1% bleach for 10 min and rinsed for 5 min by submersion in autoclaved water. The leaves were placed in lidless PCR tubes with 200 µL of either the FANA oligo targeting the *C*Las DNA Ligase gene (*LigA*) or SC-FANA (negative control). The working concentration of the FANA ASO solution was 5 µM. After wrapping the tubes tops with Parafilm, the leaves were placed under artificial light at 28 °C, a light dark cycle of 16:8 h and 75% relative humidity, in order to stimulate the absorption of the FANA ASO solutions. The tubes were filled with nuclease-free water, after the entire FANA ASO solution was absorbed by the leaves. Treated and untreated leaves were placed inside 50 mL Falcon tubes and exposed to *C*Las-infected, ~ 3 days old, *D. citri* adults (eight males, eight females) for seven days. Each treatment (*LigA*-FANA, SC-FANA, and untreated control) was replicated three times.

#### Analysis of gene expression

Total RNA extraction from untreated and treated samples (S2-wDi cells or *D. citri* adults) was performed using Direct-zol RNA MiniPrep (Zymo Research, Irvine, CA), following the manufacturer’s instructions. The concentration and quality of RNA were measured by spectrophotometry (Nanodrop 2000; Thermo Scientific). cDNA was synthesized from total RNA (1 µg) using the High Capacity cDNA Reverse Transcription kit (Thermo Fisher Scientific). Quantitative PCR assays were conducted using a QuantStudio 6 Flex Real-Time PCR Instrument (Thermo Fisher Scientific) and the SYBR Green PCR Master Mix (Thermos Fisher Scientific).

Gene expression data were analyzed using the comparative critical threshold (ΔΔCt) method^47^, in which the expression of the target mRNA in FANA-treated samples was compared to its expression in untreated samples. The wDi gene *wsp*, which codes for a surface protein, and the *C*Las 16S ribosomal RNA gene were used as an internal control for cell culture and insect bioassays, respectively, as previously described by Sandoval-Mojica et al.^48^ Primers were designed for the target and the reference genes (Supplementary Table S1) using Primer3 v. 0.4.0 software^49^. Efficiency of target and reference gene amplification was confirmed to be within a range of 90–110% for all qPCR assays.

The Shapiro–Wilk normality test and the Levene test of homogeneity of variances were employed to determine the type of distribution for the data obtained in each treatment. *T-tests* for independent samples or Mann–Whitney *U*-tests, depending on data distribution, were used to test for significant differences in expression levels (ΔCt values) of the target genes between the experimental and control conditions. *P*-values less than 0.05 were considered to be statistically significant. The software STATISTICA 13.3 (TIBCO Software Inc, Palo Alto, CA) was used for the data analysis**.**

### wDi viability assay

wDi cells were isolated from S2 cells following the protocol described in Gamston and Rasgon^50^. In brief, the S2 cells were lysed by vortexing the samples with sterile 3 mm borosilicate glass beads at room temperature. The supernatant was centrifuged at 2500×*g* (10 min at 4 °C), passed through a filter of 5 µm and centrifuged again at 18,000×*g* (10 min at 4 °C). The wDi pellet was resuspended in S2 complete media and finally purified using a 2.7 µm filter. Extracted wDi cells were seeded in 96-well culture dishes, at a density of 300,000 cells/mL, and incubated with 5 µM of either the FANA oligo complementary to the wDi *gryA* gene or the scramble control. Four days after treatment, 800 µL of cell suspension was split into two equivalent samples. One of them was kept untreated and the other one was added with 100 µL of PMA Enhancer for Gram Negative Bacteria (Biotium, Hayward, CA), followed by 5 µL of 2.5 mM propidium monoazide (PMAxx Dye; Biotium). The samples were covered in aluminum foil and incubated for 10 min on a rocker at room temperature. Subsequently, the samples were exposed to intense visible light for 15 min in order to crosslink the PMA with the bacterial dsDNA (PMA-Lite; Biotium). The bacterial cells were pelleted by centrifugation at 5000×*g* for 10 min and used for DNA isolation (DNeasy Blood & Tissue Kit; Qiagen, Valencia, CA). For dead cell control samples, 800 µL of untreated wDi suspension was heat inactivated at 95 °C for 5 min and processed as previously described.

For absolute quantification of the wDi copy number, a 250 bp DNA fragment from the wDi *gyrA* gene was amplified and purified. Ten-fold serial dilution of this fragment were used in qPCR reactions to generate a standard curve that allowed conversion of delta threshold cycle values (Ct with PMA—Ct without PMA) into an estimate of alive wDi genome copy number. Quantitative PCR assays were conducted using a QuantStudio 6 Flex Real-Time PCR Instrument and the Syber Green PCR Master Mix. The standard curve obtained for wDi was (*y* = − 3.594*x* + 3.796; *R*^2^ = 0.99). One-way analysis of variance (ANOVA) was used to evaluate the dissimilarities in wDi copy number between the treatments using the software STATISTICA 13.3. Means were separated by Tukey's honest significant difference test. *P*-values equal or less than 0.05 were considered statistically significant.

### FANA ASO targeting *C*Las in *D. citri*

A group of infected *D. citri* teneral adults (eight males, eight females) were fed an artificial diet solution, consisting of 17% (w:v) sucrose in sterile, distilled water and 0.5% green food dye. A FANA ASO targeting the *C*Las *LigA* gene was diluted into the artificial diet to a final concentration of 5 µM. The artificial feeding system consisted in a bottomless petri dish (35 mm × 10 mm), two pieces of thinly stretched Parafilm and a filter paper disc^51^; the artificial diet (300 µL) was dispensed on the filter paper located between the Parafilm layers. Feeding assays were held for seven days in an environmental incubator at 16:8 h light:dark cycle, 27 ± 2 °C, and 60–65% relative humidity. Psyllids were placed in feeding chambers containing the *LigA* FANA, untreated diet, or a 5 µM diet solution containing a FANA molecule with a random nucleotide sequence (“scramble control”) as a negative control. Three replicates were conducted for each treatment.

Insect DNA was extracted using the DNeasy Blood and Tissue Kit (QIAGEN) and its concentration quantified by spectrophotometry. DNA samples were diluted to 50 ng µL^−1^ for subsequent qPCR analysis. A multiplex *Taq*Man qPCR assay was performed using probe and primers targeting *C*Las *16S rRNA* gene and *D. citri* wingless (*Wg*) gene (Supplementary Table S1). All qPCR reactions were performed on a QuantStudio 6 Flex Real-Time PCR Instrument using the PerfeCta qPCR ToughMix, Low ROX (Quanta BioSciences, Gaithersburg, MD).

Absolute quantification of *C*Las copy number was calculated using dilution series of a plasmid containing the target region of the *C*Las *16S rRNA* gene, as described in Chu et al.^52^. The standard curve obtained for *C*Las was (*y* = − 3.286*x* + 10.338; *R*^2^ = 0.99). *C*Las copy number was divided by the *Wg* gene copy number in the same sample. The differences in *C*Las copy number between the treatments were analyzed by Kruskal–Wallis H test via STATISTICA 13.3. Medians were separated by pair-wise comparisons using Mann–Whitney *U* tests. *P*-values equal or lower than 0.05 were considered statistically significant.

### FANA ASO root infusions and *C*Las quantification *in planta*

Two to three years-old Citrumelo (*Citrus paradisi* × *Poncirus trifoliata*), not treated with systemic insecticides, were used for plant assays. The trees were inoculated with *C*Las by exposing them to infected *D. citri* for one month. After the inoculation feeding period, all developmental stages of *D. citri* were eliminated from the trees by an insecticide treatment. The plants were maintained in a greenhouse for four months to allow for systemic infection *C*Las.

The initial *C*Las titer (T0) of each tree was calculated by collecting three leaves per plant and extracting their genomic DNA, followed by quantitative real-time PCR. The *Citrus* trees were then treated with the *LigA* FANA ASO by root infusion. A single root from each tree was gently scraped under water with a razor blade and fitted into clear silicone tubing (1 m long, 6 mm diameter). The tubes were filled with 5 mL of either a 5 µM FANA ASO solution, a 5 mg/mL streptomycin sulfate solution or water (untreated control). Tubes were filled with water 24 h post treatment. From each tree, three leaves were removed from similar locations as the T0 samples at two, seven, 14- and 30-days post treatment, to monitor the effect of the FANA ASO on the plants *C*Las titer. Five trees were used per treatment.

Leaf DNA was extracted as described in Pelz-Stelinski et al.^46^. and diluted to 15 ng µL^−1^. A multiplex *Taq*Man qPCR assay was performed as stated before, using probe and primers targeting *C*Las *16S rRNA* gene and the citrus mitochondrial cytochrome oxidase gene (*Cox*) as internal control for DNA extractions (Supplementary Table S1). *C*Las copy number was quantified as previously reported^52^. The standard curve obtained for *C*Las in the plant experiments was (*y* = − 3.312*x* + 11.763; *R*^2^ = 0.99). The treatments effect was expressed as percent change in *C*Las titer [(mean titer after treatment − mean titer prior to treatment)/mean titer prior to treatment] × 100^44^. Initial and final *C*Las copy number in plants was compared by treatment using one-way analysis of variance (ANOVA). Means were separated by Fisher’s least significant difference (LSD) test, considering *P*-values equal or less than 0.05 as statistically significant.

### *C*Las transmission efficiency assay

Infected adult *D. citri* (≤ 3 days old) were placed in feeding arenas where they were exposed to a 5 µM FANA solution (*LigA*-FANA) or a 17% (w:v) sucrose solution, as described above. After seven days of continuous feeding, the psyllids were transferred to uninfected nine months old *Citrus macrophylla* plants for a 15-day transmission feeding period. Twenty adult psyllids (10 males and 10 females) were enclosed on individual plants using 1 L plastic deli containers equipped with mesh insect-proof panels for ventilation. Five plants were used per treatment. *C*Las-free psyllids were caged with healthy *Citrus macrophylla* seedlings as negative control. After the transmission feeding period, psyllids were collected and stored in 80% ethanol for subsequent *C*Las detection. The plants were maintained in an insect proof greenhouse and sampled after six months to quantify *C*Las infection. Three leaves were randomly sampled from each plant. DNA was extracted to determine the *C*Las copy number of each seedling as described before. Kruskal–Wallis H test and Mann–Whitney U test were used to compare *C*Las copy number between the treatments (*P* ≤ 0.05).

## Supplementary Information

Supplementary Table S1.

## References

[1] Batool A (2007). Citrus greening disease-a major cause of citrus decline in the world-a review. Hortic. Sci..

[2] Halbert SE, Manjunath KL (2004). Asian citrus psyllids (Sternorrhyncha: Psyllidae) and greening disease of citrus: A literature review and assessment of risk in Florida. Fla Entomol..

[3] Bové JM (2006). Huanglongbing: A destructive, newly emerging, century-old disease of citrus. J. Plant Pathol..

[4] Hall DG, Richardson ML, El-Desouky A, Halbert SE (2013). Asian citrus psyllid, *Diaphorina citri*, vector of citrus huanglongbing disease. Entomol. Exp. Appl..

[5] Baumann P (2005). Biology of bacteriocyte-associated endosymbionts of plant sap-sucking insects. Annu. Rev. Microbiol..

[6] Nakabachi A (2006). The 160-kilobase genome of the bacterial endosymbiont Carsonella. Science.

[7] Nakabachi A (2013). Defensive bacteriome symbiont with a drastically reduced genome. Curr. Biol..

[8] Werren JH, Baldo L, Clark ME (2008). *Wolbachia*: Master manipulators of invertebrate biology. Nat. Rev. Microbiol..

[9] Hoffmann M, Coy MR, Gibbard HNK, Pelz-Stelinski KS (2014). *Wolbachia* infection density in populations of the Asian citrus psyllid (Hemiptera: Liviiidae). Environ. Entomol..

[10] Tiwari S, Mann RS, Rogers ME, Stelinski LL (2011). Insecticide resistance in field populations of Asian citrus psyllid in Florida. Pest Manag. Sci..

[11] Qureshi JA, Kostyk BC, Stansly PA (2014). Insecticidal suppression of Asian citrus psyllid *Diaphorina citri*(Hemiptera: Liviidae) vector of Huanglongbing pathogens. PLoS ONE.

[12] Grafton-Cardwell EE, Stelinski LL, Stansly PA (2013). Biology and management of Asian citrus psyllid, vector of the huanglongbing pathogens. Annu. Rev. Entomol..

[13] Kanga LHB, Eason J, Haseeb M, Qureshi J, Stansly P (2016). Monitoring for insecticide resistance in Asian Citrus Psyllid (Hemiptera: Psyllidae) populations in *Florida*. J. Econ. Entomol..

[14] Chen XD, Gill TA, Ashfaq M, Pelz-Stelinski KS, Stelinski LL (2018). Resistance to commonly used insecticides in Asian citrus psyllid: Stability and relationship to gene expression. J. Appl. Entomol..

[15] Tian F, Mo F, Rizvi SAH, Li C, Zeng X (2018). Detection and biochemical characterization of insecticide resistance in field populations of Asian citrus psyllid in Guangdong of China. Sci. Rep..

[16] Douglas AE, Prosser WA (1992). Synthesis of the essential amino acid tryptophan in the pea aphid (*Acyrthosiphon pisum*) symbiosis. J. Insect Physiol..

[17] Machado-Assefh CR, Lopez-Isasmendi G, Tjallingii WF, Jander G, Alvarez AE (2015). Disrupting *Buchnera aphidicola*, the endosymbiotic bacteria of *Myzus persicae*, delays host plant acceptance. Arthropod Plant Interact..

[18] Killiny N, Hajeri S, Tiwari S, Gowda S, Stelinski LL (2014). Double-stranded RNA uptake through topical application, mediates silencing of five CYP4 genes and suppresses insecticide resistance in *Diaphorina citri*. PLoS ONE.

[19] Kishk A (2017). RNA interference of carboxyesterases causes nymph mortality in the Asian citrus psyllid, *Diaphorina citri*. Arch. Insect. Biochem. Physiol..

[20] Galdeano DM, Breton MC, Lopes JRS, Falk BW, Machado MA (2017). Oral delivery of double-stranded RNAs induces mortality in nymphs and adults of the Asian citrus psyllid, *Diaphorina citri*. PLoS ONE.

[21] Kole R, Krainer AR, Altman S (2012). RNA therapeutics: Beyond RNA interference and antisense oligonucleotides. Nat. Rev. Drug Discov..

[22] Kalota A (2006). 2’-Deoxy-2’-Fluoro-β-D-Arabinonucleic Acid (2’F-ANA) modified oligonucleotides (ON) effect highly efficient, and persistent, gene silencing. Nucleic Acids Res.

[23] Ferrari N (2006). Characterization of antisense oligonucleotides comprising 2’-Deoxy-2’-Fluoro-β-d-Arabinonucleic Acid (FANA). Specificity, potency, and duration of activity. Ann. N. Y. Acad. Sci..

[24] Meister G, Tuschl T (2004). Mechanisms of gene silencing by double-stranded RNA. Nature.

[25] Mello CC, Conte DC (2004). Reviling the world of RNA interference. Nature.

[26] Liang XH, Sun H, Nichols JG, Crooke ST (2017). RNase H1-dependent antisense oligonucleotides are robustly active in directing RNA cleavage in both the cytoplasm and the nucleus. Mol. Ther..

[27] Lok CN (2002). Potent gene-specific inhibitory properties of mixed-backbone antisense oligonucleotides comprised of 2'-deoxy-2'-fluoro-d-arabinose and 2'-deoxyribose nucleotides. Biochemistry.

[28] Min KL (2002). Oligonucleotides comprised of alternating 2'-deoxy-2'-fluoro-beta-d-arabinonucleosides and D-2'-deoxyribonucleosides (2'F-ANA/DNA 'altimers') induce efficient RNA cleavage mediated by RNase H. Bioorg. Med. Chem. Lett..

[29] Moroz E (2016). Carrier-free gene silencing by amphiphilic nucleic acid conjugates in differentiated intestinal cells. Mol. Ther. Nucleic Acids..

[30] Watts JK, Katolik A, Viladoms J, Damha MJ (2009). Studies on the hydrolytic stability of 2’-fluoroarabinonucleic acid (2’F-ANA). Org. Biomol. Chem..

[31] Shen X, Corey DR (2018). Chemistry, mechanism and clinical status of antisense oligonucleotides and duplex RNAs. Nucl. Acids Res..

[32] Christiaens O, Smagghe G (2014). The challenge of RNAi-mediated control of hemipterans. Curr. Opin. Insect Sci..

[33] Allen ML, Walker WB (2012). Saliva of *Lygus lineolaris* digests double stranded ribonucleic acids. J. Insect. Physiol..

[34] Christiaens O, Swevers L, Smagghe G (2014). DsRNA degradation in the pea aphid (*Acyrthosiphon pisum*) associated with lack of response in RNAi feeding and injection assay. Peptides.

[35] Kois P (1993). Synthesis and some properties of modified oligonucleotides. II. Oligonucleotides containing 2’-Deoxy-2’-Fluoro-β-d-Arabinofuranosyl pyrimidine nucleosides. Nucl. Nucleot..

[36] Noronha AM (2000). Synthesis and biophysical properties of Arabinonucleic acids (ANA): Circular dichroic spectra, melting temperatures, and ribonuclease H susceptibility of ANA-RNA hybrid duplexes. Biochemistry.

[37] Souleimanian N (2012). Antisense 2’-Deoxy-2’-Fluoro-β-D-Arabinonucleic Acid (2’F-ANA) oligonucleotides: In vitro gymnotic silencers of gene expression whose potency is enhanced by fatty acids. Mol. Ther. Nucleic Acids.

[38] Lebedeva I, Benimetskaya L, Stein CA, Vilenchik M (2000). Cellular delivery of antisense oligonucleotides. Eur. J. Pharm. Biopharm..

[39] Ammar ED, Shatters RG, Hall D (2011). Localization of *Candidatus* Liberibacter asiaticus, associated with citrus Huanglongbing disease, in its psyllid vector using fluorescence *in situ* hybridization. J. Phytopathol..

[40] Ammar ED, Shatters RG, Lynch C, Hall DG (2011). Detection and relative titer of *Candidatus* Liberibacter asiaticus in the salivary glands and alimentary canal of *Diaphorina citri* (Hemiptera: Psyllidae) vector of citrus Huanglongbing disease. Ann. Entomol. Soc. Am..

[41] Ghanim M (2017). *Candidatus* Liberibacter asiaticus accumulates inside endoplasmic reticulum associated vacuoles in the gut cells of *Diaphorina citri*. Sci. Rep..

[42] Boina DR, Bloomquist JR (2015). Chemical control of the Asian citrus psyllid and of huanglongbing disease in citrus. Pest Manag. Sci..

[43] Hoffmann M (2013). Heat treatment eliminates ‘*Candidatus* Liberibacter asiaticus’ from infected citrus trees under controlled conditions. Phytopathology.

[44] Zhang M (2014). Effective antibiotics against ‘*Candidatus* Liberibacter asiaticus’ in HLB-affected citrus plants identified via the graft-based evaluation. PLoS ONE.

[45] Hu J, Jiang J, Wang N (2018). Control of citrus Huanglongbing via trunk injections of plant defense activators and antibiotics. Phytopathology.

[46] Pelz-Stelinski KS, Brlansky RH, Ebert TA, Rogers ME (2010). Transmission parameters for *Candidatus* Liberibacter asiaticus by Asian citrus psyllid (Hemiptera: Psyllidae). J. Econ. Entomol..

[47] Livak KJ, Schmittgen D (2001). Analysis of relative gene expression data using real-time quantitative PCR and the 2^- ΔΔCt^ method. Methods.

[48] Sandoval-Mojica AF, Altman SA, Hunter WB, Pelz-Stelinski KS (2020). Peptide conjugated morpholinos for management of the huanglongbing pathosystem. Pest Manag. Sci..

[49] Untergasser A, Cutcutache I, Koressaar T, Ye J, Faircloth BC, Remm M, Rozen SG (2012). Primer3-new capabilities and interfaces. Nucleic Acids Res.

[50] Gamston C, Rasgon J (2007). Maintaining *Wolbachia* in Cell-free Medium. J. Vis. Exp..

[51] Russell CW, Pelz-Stelinski KS (2015). Development of an artificial diet and feeding system for juvenile stages of the Asian citrus psyllid, *Diaphorina citri*. Entomol. Exp. Appl..

[52] Chu CC, Gill TA, Hoffmann M, Pelz-Stelinski KS (2016). Inter-population variability of endosymbiont densities in the Asian citrus psyllid (*Diaphorina citri* Kuwayama). Microb. Ecol..

