# Supplementary material for: Antibacterial FANA oligonucleotides as a novel approach for managing the Huanglongbing pathosystem
Source: Sci Rep. 2021 Feb 2;11:2760. doi: 10.1038/s41598-021-82425-8 (PMC7854585; doi:10.1038/s41598-021-82425-8)
Supplement: Supplementary file 1 — Supplementary Table S1. [file 41598_2021_82425_MOESM1_ESM.docx]

**Antibacterial FANA oligonucleotides as a novel approach for managing the Huanglongbing pathosystem**

Andrés F. Sandoval-Mojica, Wayne B. Hunter, Veenu Aishwarya, Sylvia Bonilla and Kirsten S. Pelz-Stelinski

**Supplementary Table S1. qPCR primers used in analysis of gene expression.**

| . Target species | Target gene | PRIMER/**PROBE**  SEQUENCE (5’- 3’) |  |
| --- | --- | --- | --- |
| ***Wolbachia*** | ***gyrA*** | ACTAACATCATGCCGCTTCC  ATTGACGCACATCGCTTACA | |
| ***Wolbachia*** | ***wsp*** | AGGGCTTTACTCAAAATTGG  CACCAACGTATGGAGTGATAGG | |
| ***Candidatus* Liberibacter asiaticus** | ***LigA*** | ATTCCAATCGAGGCATTGAG  TCAGAGCGTGCTAAATCAGG | |
| ***Candidatus* Liberibacter asiaticus** | ***16SrRNA*** | TCGAGCGCGTATGCAATACG  GCGTTATCCCGTAGAAAAAGGTAG  **AGACGGGTGAGTAACGCG** | |
| ***Diaphorina citri*** | ***Wingless* (*Wg*)** | GCTCTCAAAGATCGGTTTGACGG  GCTGCCACGAACGTTACCTTC  **TTACTGACCATCACTCTGGACGC** |  |
| ***Citrus spp*** | ***Cox*** | GTATGCCACGTCGCATTCCAGA  GCCAAAACTGCTAAGGGCATTC  **ATCCAGATGCTTACGCTGG** |  |
|  |  |  |  |
|  |  |  |  |
